# Individual predictors of frequent emergency department use: a scoping review

**DOI:** 10.1186/s12913-016-1852-1

**Published:** 2016-10-20

**Authors:** Cynthia Krieg, Catherine Hudon, Maud-Christine Chouinard, Isabelle Dufour

**Affiliations:** 1 Faculty of Medicine and Health Sciences, Université de Sherbrooke, 1500 James-Quintin, app. 3001, Sherbrooke, Québec J1E 0E5 Canada; 2 Department of Family Medicine and Emergency Medicine, Université de Sherbrooke, Sherbrooke, Québec Canada; 3 Centre hospitalier universitaire de Sherbrooke, Sherbrooke, Québec Canada; 4 Département des sciences de la santé, Université du Québec à Chicoutimi, Chicoutimi, Québec Canada

**Keywords:** Emergency department, Frequent ED use, Chronic frequent ED use

## Abstract

**Background:**

A small proportion of patients use an excessively large amount of emergency care resources which often results in emergency department (ED) overcrowding, decreased quality of care and efficiency. There is a need to better identify these patients in order to target those who will benefit most from interventions adapted to their specific needs. We aimed to identify the predictive factors of short-term frequent use of ED (over a 1-year period) and chronic frequent use of ED (over a multiple-year period) and to highlight recurring characteristics in patients.

**Methods:**

A scoping review was performed of all relevant articles found in Medline published between 1979 and 2015 (Ovid). This scoping review included a total of 20 studies, of these, 16 articles focussed on frequent ED users and four others on chronic frequent ED users.

**Results:**

A majority of articles confirm that patients who frequently visit the ED are persons of low socioeconomic status. Both frequent and chronic frequent ED users show high levels of health care use (other than the ED) and suffer from multiple physical and mental conditions.

**Conclusions:**

This research highlights which individual factors predict frequent emergency department use. Further research is needed to better characterize and understand chronic frequent users as well as the health issues and unmet medical needs that lead to chronic frequent ED use.

## Background

A small proportion of patients utilize a disproportionately large amount of acute emergency care resources [1]. They represent as little as 2.7 % [2] of patients attending the emergency department (ED), but make up to 67 % [3] of all ED visits over a given period of time (usually 1 year). Many studies have discussed the concept of frequent users and their multiple characteristics. A recurring definition is four ED visits or more during a 12-month period [4–11].

Frequent ED visits represent substantial costs to the health care system [12–14]. They also decrease ED efficiency [15], contribute to ED overcrowding [16, 17] and can potentially impact services by redirecting them away from urgent cases [13]. Quality of care received may be suboptimal for frequent ED users, as care can be fragmented, episodic and poorly coordinated [18–21]. Physicians could also hold biases and feel less empathy for frequent ED patients [22]. Hence, the use of ED services by frequent users can often be perceived as inappropriate and nonurgent [3, 23]. As a result, the uncoordinated acute care received in the ED by these patients can be less effective compared to what they receive or would receive in primary care [24–26].

Previous studies have shown that frequent ED users are more likely to have chronic diseases, suffer from mental illnesses or have substance use disorders [4, 5, 27–32]. It has also been observed that from 1 year to the next, there is a natural decline of ED use by frequent users. However, the attrition rates of those who remained frequent ED users over the years decreased [30], making them an ideal group for targeted interventions. Very few studies have examined predictors of chronic frequent ED use, whereas frequent ED use is regarded as an excessive number of visits during a single year, chronic frequent ED use is defined as frequently visiting the ED for multiple years in a row. Improving our understanding of the needs of frequent and chronic frequent ED users and defining a new approach to correctly identify these patients would allow healthcare professionals to intervene before frequent use occurs and redirect them to more appropriate health services [33].

The main objective of this scoping review was to identify predictive factors of frequent use found in the literature and to highlight trends. A secondary objective was to underline the factors that are predictive of chronic frequent use over a multiple-year period.

## Methods

We used the methodological framework for conducting a scoping review developed by Arksey and O’Malley [34]. Scoping review is recognized as a process of mapping the main concepts of a research area to its source and evidence available in the literature. The five key phases the authors developed were followed in order to maintain a rigorous and transparent method for data collection, analysis and interpretation: 1) identifying the research question; 2) identifying relevant studies; 3) selecting studies; 4) charting the data; and 5) collating, summarizing, and reporting the results.

### Scoping review

1. Identifying the research question
  Based on current knowledge found in an initial review of the literature, our primary research question was defined as follows:
  Which factors are predictive of frequent emergency department use?
  A secondary question was also explored:
  Which factors predict chronic frequent emergency department use over a multiple-year period?
2. Identifying relevant studies
  An electronic literature search of Medline (Ovid) for English and French articles published between 1979 and May 2015 was conducted. No articles discussing frequent or chronic frequent ED use were found prior to 1979. The following key words were used: Frequent user, Frequent attender, Heavy user, Super user, Repeat user and Emergency department. One hundred and forty articles were found. In addition to the primary search, an examination of the reference list of two systematic reviews, one literature review (found during the primary search) and the articles included in the review was done (hand searching). The latest systematic review published focused on frequent users and callers to emergency medical services [15]. Most of the studies also focused on case management, analyzed specific populations such as elderly people, mentally ill patients or 911 callers, or used descriptive statistics to define frequent ED users. Only one of the studies found in this paper was used in our scoping review. The second systematic review, which was published 5 years ago, only included American studies [35]. Finally, the literature review examined gaps in current knowledge and stated efforts needed in order to identify factors predictive of future frequent ED use before it occurs. Our scoping review adds new information as it provides an international perspective of all studies that have rigorously analysed predictive factors of heavy ED use.
3. Selecting studies (Fig. 1)
  In order to be included in the review, studies had to 1) report frequent ED use in adult populations, 2) define frequent use as a minimum of 3 or more ED visits per year and 3) use regression methods to define predictive factors of frequent ED use. Studies limited to a specific population like psychiatric, geriatric, homeless or addicted patients were excluded.

**Fig. 1:**
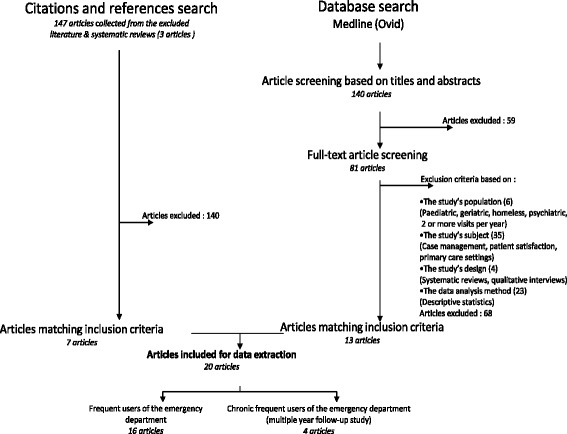
Flow chart of literature search indicating exclusion criteria and the number of included articles. Figure 1 provides the literature search process and exclusion criteria established to select final articles included for data extraction.
  The first step was to read the titles and abstracts of all potential articles and to exclude non-eligible articles based on the inclusion and exclusion criteria. This part was accomplished by one team member (CK). In case of uncertainty, the full articles were retrieved and also read by a second team member (CH). During this first step, a total of 59 articles were excluded as they had no connection with frequent ED use. Eighty-one articles from the primary search were retained for detailed evaluation. These articles were reviewed by two team members (CK and CH). Of these 81 articles, 68 were excluded: six were limited to a specific population; 35 focused on case management, primary care or patient satisfaction; four were either systematic reviews or qualitative interviews; and 23 of them used descriptive statistics to present the characteristics of frequent ED users. Another seven articles, which were all thoroughly examined by both team members (CK and CH), were retrieved by hand searching and added to the list of included articles. A total of 20 articles were included in this scoping review.
4. Charting the data
  The charting involved the extraction of information from individual articles. We collected descriptive characteristics such as authors, year of publication, study period, country where the study was held, study design, study population, sample size, definition of frequent use and predictive factors of frequent ED use.
5. Collating, summarizing, and reporting the results

## Results

### Study designs and definitions (Table 1)

Out of the 20 articles that fulfilled the inclusion criteria, five were published between 2010 and 2014 [6, 11, 28, 36, 37]. All the other studies were published between 1998 and 2009 [3–5, 8–10, 23, 30, 33, 38–42] with the exception of one that was published in 1987 [31].

**Table 1:** Characteristics of included studies

Reference	Authors	Year of publication	Study period	Country	Study design	Population	Sample size	Definition
[7]	Andrén et al.	1987	3 years (October 1979 through October 1982)	Sweden	Prospective cohort study	Cohort of frequent users of the St Göran’s Hospital ED	232	4 or more visits to the ED during the index year
[38]	Rask et al.	1998	24 months (1992–1994)	USA	Cohort observational study	Random sample of adults visiting a public hospital in Atlanta, Georgia	351	More than 10 ED visits during the 2-year follow-up period
[30]	Mandelberg et al.	2000	5 years (July 1 1993 to June 30 1998)	USA	Cross-sectional and retrospective cohort study	Database of all 348 858 visits made to the San Francisco General Hospital ED during the study period	43,383	5 or more visits in a 12 month period
[5]	Hansagi et al.	2001	1 year (January 1 to December 31 1996)	Sweden	Retrospective database study	Frequent and infrequent users who visited the Huddinge Hospital ED during the study period	47,349	4 or more visits per year
[39]	Okuyemi et al.	2001	3 years (July 1 1993 to June 30 1996)	USA	Retrospective database review	Frequent and infrequent ED users of a university hospital	12,258; 13,387; 13,219	3 or more visits per year
[10]	Huang et al.	2003	1 year (October 1 2000 to September 30 2001)	Taiwan	Retrospective study (telephone interviews)	Frequent and infrequent ED users randomly selected in a medical center	800	4 or more visits per year
[8]	Sun et al.	2003	5 months (February through June 1995)	USA	Cross-sectional multicenter ED survey	Adult patients who came to the ED with selected problems	2333	4 or more self-reported prior ED visits
[23]	Ruger et al.	2004	1 year (January 1 2001-December 31 2001)	USA	Retrospective cross-sectional study	All ED visits to an urban academic hospital	71,941	Group 1: one ED visit in 2001, group 2: two visits, group 3: three to five visits, group 4: six to 20 visits, and group 5: more than 20 visit
[40]	Zuckerman et al.	2004	2 years (1997 and 1999)	USA	National Survey data review	1997 and 1999 National Survey of America’s Families	89,626	3 or more visits per year
[41]	Freitag et al.	2005	1 year	USA	Data from 2 randomized controlled trials	Patients with chronic daily headache (>15 headache days per month) with at least one ED visit	785	3 to 6 ED visits per year
[3]	Griswold et al.	2005	6 years (1996 to 2001)	USA	Data from four prospective cohort studies	Adults presenting with acute asthma to 83 US EDs	3151	6 or more ED visits per year
[4]	Hunt et al.	2006	1 year (July 2000 through June 2001)	USA	Population-based Community Tracking Study Household Survey	Households in 60 randomly selected communities and in a national supplemental sample	49,603	4 or more visits in a single year
[33]	Pines et al.	2006	3 months (July through September 2004)	USA	Retrospective cohort study	Asthmatics in Southeastern Pennsylvania	1799	3 or more visits in a 12 month period
[42]	Moore et al.	2007	24 months (January 1 2003 through December 31 2004)	Australia	Retrospective cohort study	All patients who attended the ED during the study period	40,942	Re-presentation to the ED within 28 days of discharge
[9]	Friedman et al.	2009	3 years (2004–2006)	USA	Longitudinal population-based survey	Randomly selected severe headache sufferers	13,451	4 or more ED visits for headhache treatment in the previous 12 months
[37]	Paul et al.	2010	3 years (January 1 2005 through December 31 2007)	Singapore	Retrospective database review	Patients who attended the ED from 1 January-31 December 2006 without prior attendance during the 12 months were tracked for 12 months	82,172	5 or more visits to an ED during the last 12 months
[6]	Bieler et al.	2012	1 year (April 2008-March 2009)	Switzerland	Retrospective chart review case-control	Randomized samples of frequent and nonfrequent users of the Lausanne University Hospital	719	4 or more visits to an ED during the last 12 months
[27]	Doupe et al.	2012	1 year (Fiscal year 2004–2005)	Canada	Retrospective health record review	All Manitobans with at least 1 ED visit in the Winnipeg Health Region	105,687	7 to 17 ED visits per year
[36]	Billings et al.	2013	6 years (2004–2009)	USA	Prospective predictive modeling	Medicaid ED users in New York City	205,139	Multiple subgroups (see article)
[11]	Palmer et al.	2014	1 year (2009)	Canada	Retrospective database review	All ED visits during 1 calendar year to an urban regional hospital, an urban urgent care centre and a rural community hospital	59,803	4 or more visits to an ED in a year

Twelve studies were from the USA [3, 4, 8, 9, 23, 33, 38–41], two were from Canada [11, 27], two were from Sweden [5, 7] and the four other studies were from Singapore [37], Switzerland [6], Taiwan [10] and Australia [42].

There was no standard definition of a frequent user, although the majority of the included studies (8) defined frequent use as 4 or more ED visits during a 12-month period [4–11]. Other definitions varied between 3 [39] and 17 [27] or more ED visits per year. A total of eight studies analyzed frequent ED use during a multiple-year period [7, 9, 30, 36, 38–40, 42]. However, only four [7, 36, 38, 39] of these studies used regression modeling to identify factors predicting chronic frequent ED use. Of the observed populations, frequent ED users represented between 3.5 % [10] and 29 % [36] of all patients attending the ED but accounted for 12.1 % [30] to 67 % [3] of all ED visits made.

### Characteristics of frequent ED use (Table 2)

#### Gender

Three studies [27, 30, 37] found that males were more likely to be frequent users. Another study [11] found that being a female was predictive of frequent ED attendance and that male patients had a lower likelihood of being frequent attenders. A total of four other studies [3, 9, 33, 42] also analyzed gender as a potential independent factor but found that it was not a significant predictor of frequent ED use.

**Table 2:** Predictive characteristics of frequent ED use

Predictive factors	Details	References
Demographic		
Male		[27, 30, 37]
Female		[11]
Age	75 years and older	[11, 37]
	Between 30 and 59 years old	[30]
	Lower age	[41]
Location	Attendance at a rural ED	[11]
	Urban area (Philadelphia County)	[33]
	Core area patients (Winnipeg Health Region)	[27]
Distance to ED	Less than 10 km	[6]
	More than 2 km	[37]
Socioeconomic		
Education	No high school diploma	[40]
	High school education (or less)	[8, 33]
Family status	Single parents	[8, 40]
	Single	[8]
	Divorced	[8]
	Being under guardianship	[6]
	Number of children living in the house	[33]
Housing status	Homeless	[30, 42]
Income	Living in lowest income areas	[27]
	Being unemployed or dependant of government welfare	[6]
	Receiving government pension	[42]
	Family income below the poverty threshold	[4]
	Low socioeconomic status	[9]
	Low income groups	[40]
	Income of less than 10 000$	[8]
Insurance	Being uninsured	[3, 4, 6]
	Medicaid	[3, 4]
	Medicare coverage	[4, 23]
	Publicly insured	[3, 40]
	Medical Assistance	[33]
	Medi-Cal sponsored	[30]
Current healthcare use	Multiple visits to a specialist physician	[9, 27, 41]
	Multiple visits to a primary care provider	[5, 8, 27, 40]
	Calling a health helpline	[27]
	Being hospitalized	[3, 5, 6, 8, 10, 27, 41]
	Outpatient visits	[4, 5, 10]
	Visiting a clinic	[4, 6, 33]
	History of past emergency department use	[3, 27, 39]
	Identifying an ED or hospital clinic as primary care site	[8]
	Prescription medication use	[3, 9, 33]
Access to primary healthcare services	Having a primary care provider	[8, 11]
	ED as primary source of care	[4, 8]
	Having another regular source of care	[10]
Medical		
Mental illness	Substance abuse problems	[10, 27, 30]
	Mental disorder	[4, 6, 8, 9, 42]
Physical disease		
Chronic condition		[10, 27]
Exacerbation of chronic conditions	Sickle cell anemia, renal failure, chronic obstructive pulmonary disease	[30]
Fair/poor physical health		[4, 40]
Other diseases	Pulmonary disease	[3, 8, 10, 37]
	Cardiovascular disease	[10]
	Gastrointestinal disease	[10]
	Cancer	[10]
Medical scores	Severe rating on MIDAS	[9, 41]
	Lower role physical domain	[41]
	Higher DRG severity score	[23]

#### Age

In a population of 59,803 patients, patients in the geriatric age group (75 and older) were more likely to be frequent ED users compared to younger adults (20 to 49 years old). They also found that, compared to young adults, the odds of frequent use were lower for older adults (50 to 74 years old) [11]. Another study [37] also found that being 75 years old or more was a predictive factor of frequent ED use. However, two other studies stated otherwise as they found that frequent ED users were more likely to be between 30 and 59 years old and significantly younger than the non-ED users [30, 41]. Conversely, six studies stated that patient age was not a significant risk factor of frequent ED use [3, 9, 10, 27, 33, 42].

#### Location

One American and two Canadian studies suggested that hospital location could be a predictive factor of frequent ED use. One study concluded that frequent ED use was predicted by attending a rural ED [11] whereas the two other studies stated that patients living in urban areas were at higher risk of becoming frequent ED users [27, 33]. One study showed that living close to the ED (less than 10 km away) increased the risk of frequent ED use [6], while another one found that traveling more than 2 km in order to get to the ED significantly increased the odds of frequent ED attendance [37].

#### Education and socioeconomic factors

Two studies stated that lower-level education was predictive of frequent ED use [8, 33] and one study suggested that those with at least a college degree were less likely to be heavy ED users [40]. Two other studies found that patient education was not a significant factor [3, 10].

Many studies concluded that patients who had a low income [4, 8, 27, 40], were unemployed [6] or received a government pension [6, 42] were more likely to be frequent ED users. A few studies, however, found that monthly household income, financial barriers [10] and poverty [33] were not significant predictors of frequent ED use. Six different studies found that the type of health insurance a patient had would potentially predict ED use [3, 4, 23, 30, 33, 40]. Three studies also noted that without health insurance patients were also more likely to fall into this category [3, 4, 6].

#### Factors associated with current healthcare use

Frequent ED users tended to heavily use other medical services; some of the variables used to describe their health care use included multiple visits to a specialist physician [9, 27, 41] multiple visits to a primary care provider [5, 8, 27, 40], calling health helplines, [27] being previously hospitalized [3, 6, 8, 10, 41], having outpatient visits [4, 10], visiting a clinic [6, 10], having a history of past ED use [39], identifying an ED or hospital clinic as primary care site [8] and finally, using prescription medication [3, 9, 33]. Accessibility to primary healthcare was also discussed in a few studies. Two studies found that frequent ED users were significantly more likely to have listed primary care providers [8, 11].

#### Medical factors

Multiple studies stated that physical diseases were an important contributing factor in heavy ED use [3, 4, 8, 10, 27, 30, 37, 40]. Frequent ED users suffered from various medical conditions which included chronic diseases [10, 27], pulmonary diseases [3, 8, 10, 37] such as asthma [8, 27], chronic obstructive pulmonary disease [37] and acute respiratory infection [37], cardiovascular diseases [10] such as heart failure [41] and stroke [27], gastrointestinal diseases [10], diabetes [27], cancer [10], and exacerbation of chronic conditions including sickle cell anemia, renal failure and COPD [30]. Poor physical health was also a predictor of ED use in two studies which measured physical health with the SF-12 Health survey [4] and with self-reported statements [10].

ED patients with mental illnesses were also at higher risk of becoming frequent ED users [4, 6, 8–10, 27, 30, 42]. The relative risk of frequent use was higher in patients who were seen for substance abuse problems such as alcohol withdrawal, alcohol dependence and alcohol intoxication [30] or abuse problems [4]. Other studies also found that patients with poor mental health [4], high ratings of psychological distress [8] and depression [9] had greater odds of being frequent ED users.

### Characteristics of chronic frequent ED use (Table 3)

Four studies examined factors that could predict frequent ED use over a multiple-year period. A first study found that previous ED visits was a predictive factor of chronic ED use. More so, they found other predictive factors such as having contact with psychiatric care, living alone and perceived loneliness [7]. In the second study, the authors used predictive modeling using standard regression techniques to predict which patients were most likely to become frequent ED users. Amongst the patients selected in the model, 77.9 % of chronic frequent users had higher levels of chronic illness and one half had multiple chronic conditions. A majority of this population also had behavioural health problems with 58.8 % of them having a history of substance abuse, 72.3 % a history of mental illness and 48.9 % having a history of both these conditions. Finally, those identified by this model had more ambulatory visits and were also at higher risk of becoming chronic users or “serial users” [36]. The third study found that patients who had at least one previous hospitalisation and at least one primary care visit were more likely to present frequent chronic use of the ED [38]. In the fourth and last study, the authors concluded that being a frequent user over the previous year was the only independent predictor of the level of ED use the following year [39].

**Table 3:** Predictive characteristics of chronic frequent ED use

Reference	Authors	Definition of chronic use	Prevalence % of patients (% of total ED visits)	Predictive factors
[7]	Andrén et al.	4 or more ED visits per year over a 3 year period	Year 1 : 31 % Year 2 : 19 %	Previous ED visits, contact with psychiatric care, living alone and perceived loneliness
[36]	Billings et al.	5 or more ED visits per year over a 3 year period	1.2 % (10,9 %)	77,9 % had higher levels of chronic illnesses 58,8 % had a history of substance abuse 72,3 % had a history of mental illness48, 9 % had a history of both substance abuse and mental illness^a^
		5 or more ED visits per year over a 5 year period	0.8 % (8,4 %)	
		3 or more ED visits per year over a 3 year period	3.5 % (19,2 %)	
		3 or more ED visits per year over a 5 year period	1.7 % (12,1 %)	
[38]	Rask et al.	More than 10 subsequent ED visits (2 year period)	16,6 % (65,5 %)	At least one hospitalization and at least one primary care visit
[39]	Okuyemi et al.	3 or more ED visits per year over a 3 year period	9 to 11 % (between 25 and 30 %)	Being a frequent ED user during the previous year is an independent predictor of frequency of use during the following year

^a^All predictive factors found using regression model for patients visiting the ED 3 or more times during the index year

## Discussion

This scoping review aimed to conduct a scan of the current knowledge on predictive characteristics of both frequent and chronic frequent ED use. In general, patients frequently visiting the ED had a low socioeconomic status, high levels of health care use (other than the ED) and suffered from multiple physical and mental conditions. Although, to date, only a few studies have analyzed chronic frequent ED use, most predictive factors found for this population are similar to those found in frequent ED users.

In many cases, frequent use of ED is considered inappropriate [3, 4, 23]. As a result, the uncoordinated acute care received in the ED by these patients is potentially less effective compared to what they would receive in primary care. Frequent ED users also seek a lot of medical help outside of the ED, suggesting that they may have unmet healthcare and medical needs [8]. Most of these frequent users suffer from chronic conditions, many of which are ambulatory care sensitive conditions (ACSC), such as asthma [8, 27] COPD [37], and cardiovascular diseases like diabetes [29] and heart failure [37]. These are chronic diseases for which adequate ambulatory primary care could prevent deterioration or complications requiring ED visits or hospitalizations [43]. For some patients suffering from ACSC, the interaction with psychological and/or social problems could add a level of complexity that would interfere with usual care and lead to unmet healthcare needs [44, 45]. These predictive factors portray the complexity and multifaceted needs of frequent ED users. In order to provide adequate new strategies to better meet these patients’ healthcare needs, all of these factors must be taken into account.

The Agency for Healthcare Research and Quality Multiple Chronic Conditions Research Network defines complexity as the gap between an individual’s needs and the capacity of health services to answer those needs [46]. The implicit objective of the healthcare system is to identify these individuals’ needs and to treat them accordingly. The more complicated their needs get, the harder it is for healthcare services to provide the appropriate treatment. However, these patients often try in vain to handle their unmet healthcare needs by using multiple medical services such as the ED. Inevitably, this results in considerable costs to the healthcare system. Even though these patients try to handle their conditions, they still present low health indicators such as high mortality rates [47].

Hence, frequent ED users rely heavily on the ED for ambulatory care; however they could greatly benefit from timely access to preventive and continuous care in a primary care setting. Case management is recognized internationally as an appropriate intervention strategy for complex health situations and to improve the capacity of healthcare services to answer these patients’ particular needs [48, 49].

Due to the small number of studies analysing factors predictive of chronic frequent ED use, it is a challenge to properly portray these ED patients and to distinguish them from frequent ED users. Chronic frequent users share similar traits with frequent ED users, such as heavy use of health care services other than the ED. They also suffer from similar physical and mental diseases. However, more research is needed to better understand the underlying reasons leading to frequent users becoming chronic frequent users over time.

## Strengths and limitations

To our knowledge, this is the first paper to review and summarise predictive factors of frequent and chronic ED use analyzed by regression modelling. Two independent authors reviewed all articles. However, only English and French articles were included, which may lead to selection bias. Furthermore, a majority of articles were based in American settings, limiting the comparability of results to other countries around the world. The lack of consistent definition of frequent ED use does not allow strong comparisons among studies. Only one database (Medline) was used in order to find relevant articles and this study did not include a quantitative summary of the results found. A meta-analysis could be the next step in research on predictive characteristics of frequent and chronic frequent ED users. The influence of the Patient Protection and Affordable Care Act (PPACA) on frequent ED use is yet to be known.

## Conclusions

Many frequent ED users have high levels of health care use (other than the ED), a lower socioeconomic status, and suffer often from concomitant multiple physical and mental conditions. More research is needed in order to better understand factors leading to chronic ED use and to develop effective strategies to better meet their complex health care needs.

## Abbreviations

ACSC: Ambulatory care sensitive conditions
COPD: Chronic obstructive pulmonary disease
DRG: Diagnosis-related group
ED: Emergency department
MIDAS: Migraine disability instrument
PPACA: The patient protection and affordable care act
